# Complication patterns and postoperative outcomes in surgical patients admitted to intensive care units

**DOI:** 10.2478/jccm-2025-0044

**Published:** 2025-10-31

**Authors:** Caroline Tolentino Sanches, Silvia Paulino Ribeiro Albanese, Monique Elen Robuste, Gabriela Gomes da Silva, Marcos Toshiyuki Tanita, Cintia Grion

**Affiliations:** Universidade Estadual de Londrina, Londrina, Brazil

**Keywords:** postoperative complications, in-hospital mortality, intensive care unit, clinical protocols

## Abstract

**Objective:**

To analyze the frequency and types of postoperative complications and risk factors for in-hospital mortality.

**Methods:**

This retrospective longitudinal study included adult patients who underwent surgical procedures and were admitted to the intensive care unit of a university hospital between March and July 2022. Study variables included sociodemographic, clinical, and epidemiological data; postoperative complications and hospital outcomes. The significance level was set at 5%.

**Results:**

We analyzed 202 patients, with a median age of 67 years (IQR 55–74) and a predominance of males (62.4%). Inhospital mortality was 26.2%. Postoperative complications occurred in 84.7% of patients, with cardiovascular (53.4%), infectious (49.5%), and gastrointestinal (48.5%) complications being the most frequent. Early postoperative feeding was initiated in 34.2% of cases, and a delay was associated with a higher risk of complications. Nausea and vomiting prophylaxis were administered to most patients—intraoperatively in 61.9% and postoperatively in 96%. In logistic regression analysis, female sex, urgent surgery, and higher SAPS 3 scores were identified as independent risk factors for death.

**Conclusions:**

Postoperative complications were highly prevalent and associated with an increased risk of death. Intra-operative nausea and vomiting prophylaxis and early postoperative feeding were associated with a lower frequency of complications. Identified risk factors for mortality included female sex, higher SAPS 3 scores, and urgent surgeries.

## Introduction

Postoperative complications are commonly associated with increased patient morbidity and mortality and may result in long-term sequelae and, ultimately, death [1,2]. Increased life expectancy, the presence of comorbidities, and the complexity of the surgical procedure are key factors contributing to the development of such complications [3].

A study conducted in hospitals in the United Kingdom revealed that 75.4% of deaths in the postoperative period occur among high-risk patients, although only one third of these patients were admitted to the intensive care unit (ICU) at any point during the postoperative period [4].

The high mortality rate among surgical patients is, in most cases, associated with multiple organ dysfunction syndrome (MODS) [5]. Therefore, identifying risk factors for complications and poor outcomes is essential, as this knowledge can influence decisions regarding ICU admission [5].

Among the recommendations to decrease surgical complications are restricting perioperative intravenous fluids, postoperative nausea and vomiting prophylaxis and control, shortening of preoperative fasting, and early postoperative feeding [6]. Implementing patient safety-focused programs has contributed to reduced hospital length of stay and surgical site infection rates [7], as well as hospital morbidity [8].

Accordingly, this study aimed to analyze the frequency and types of postoperative complications and the risk factors for in-hospital mortality in ICU patients.

## Methods

This study was submitted to and approved by the local Research Ethics Committee for Human Subjects under opinion no. 3.900.546; CAAE no. 28310420.6.0000.5231. The requirement for an Informed Consent Form (ICF) was waived for this research.

This was a retrospective longitudinal study using a convenience sample, including all adult patients aged 18 years or older who underwent surgical procedures and were admitted to the intensive care units (ICUs) of a university hospital in the immediate postoperative period. This retrospective cohort study was conducted at Londrina State University Hospital, in Londrina, Brazil, between March and July 2022.

**Fig. 1.**
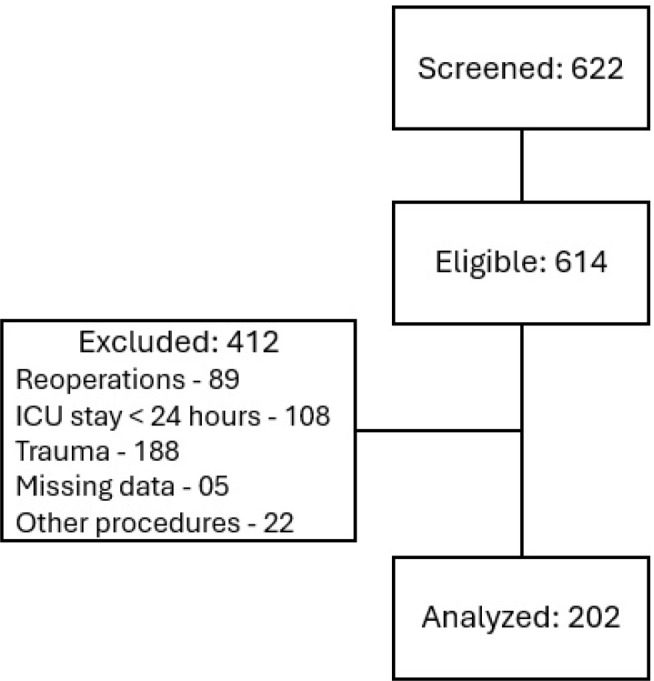
Flowchart of patients included in the study

We excluded patients if they had undergone obstetric or palliative surgeries, had experienced trauma, had reoperations during the same hospitalization after being included in the study, had undergone endovascular procedures, had ICU stays shorter than 24 hours, or had incomplete medical records (Figure 1).

Data sources for this study included patient medical records and the hospital’s electronic health information system. Data was collected using a structured form developed by researchers. The research team consisted of four nurses trained in the study subject matter to minimize collection errors.

The study variables were grouped into categories corresponding to the period from hospital admission to discharge. Variable definitions are included in supplementary Table 1.

– Sociodemographic, clinical, and epidemiological variables: age; sex; preexisting conditions (past or current use of tobacco, alcohol, or illicit drugs); comorbidities listed in the Charlson Comorbidity Index [9].
– Surgical procedure-related variables: urgency or elective status, as documented in the surgical record; contamination risk classification according to the Centers for Disease Control and Prevention (CDC) criteria [10]; surgical complexity classified as minor, moderate, or major based on fluid loss, intraoperative bleeding, and risk of cardiologic complications [11]; surgical procedure categorized according to the SUS Procedure, Medications, and Orthotics/Prosthetics Management System Table (SIGTAP), established by national Ministry of Health Ordinance No. 321/2007 [12].
– Perioperative variables: duration of preoperative fasting; type of anesthesia; use of antiemetics; use of vasoactive drugs and fluid resuscitation categorized as a maximum of 30 mL/kg according to ACERTO protocol [8], which is a local adaptation of ERAS recommendations. Time to postoperative feeding were randomly divided into time intervals, based on ERAS protocols which recommend a diet with protein be introduced early, and if tolerated, oral feeding be resumed as soon as possible.
– ICU and hospitalization-related variables: physiological variables such as vital signs, laboratory tests, and fluid balance (FB); need for oxygen therapy and mechanical ventilation (MV); timing of postoperative feeding initiation; the Simplified Acute Physiology Score 3 (SAPS 3) was assessed within the first hour of ICU admission, and the Sequential Organ Failure Assessment (SOFA) score was assessed throughout the ICU stay.
– Postoperative complications: infectious, cardiovascular, respiratory, surgical, gastrointestinal, renal, neurological, coagulation-related, and electrolyte complications.
– Outcomes: the primary outcome was in-hospital mortality; secondary outcomes included postoperative complications, ICU length of stay, and overall hospital length of stay.

The normality of the variable distribution was assessed using the Shapiro–Wilk test. Depending on the distribution, continuous variables were described as mean ± standard deviation (SD) or median and interquartile range (IQR). Student’s t-test was used to compare means of normally distributed variables with homogeneous variances, and the nonparametric Mann–Whitney U test was applied for variables with non-normal distributions and/or heterogeneous variances. Categorical variables were analyzed using the chi-square test and were presented as absolute and relative frequencies. Missing data were treated by excluding from the analysis cases with missing values in any of the variables of interest (supplementary Table 2).

The association between potential risk factors (independent variables) and the dependent variable (hospital outcome) was presented as unadjusted odds ratios (ORs) and 95% confidence intervals (95% CI), obtained through logistic regression using the Enter method (bivariate analysis). Subsequently, for multivariate analysis, logistic regression was performed using the stepwise selection method. Potential risk factors were defined based on biological plausibility. The significance level was set at 5%. Statistical analyses were performed using MedCalc® Statistical Software version 22.018 (MedCalc Software Ltd, Ostend, Belgium; https://www.medcalc.org; 2024).

## Results

During the study period, 614 surgical patients were admitted to the ICUs. Of these, 412 were excluded based on the following criteria: 89 were reoperations of patients already included in the study; 108 remained in the ICU for less than 24 hours; 188 had experienced trauma; 5 had missing medical record data that made data collection unfeasible; and 22 underwent other types of procedures (obstetric surgeries, organ procurement for donation, and wound care).

We analyzed 202 patients, with a median age of 67 years (IQR 55–74) and a predominance of males (62.4%). The most frequently reported comorbidities were hypertension (HTN), diabetes mellitus (DM), and cardiopathy. Among lifestyle factors, smoking was the most prevalent. The median ICU length of stay was 2 days (IQR 1–8), and the median hospital length of stay was 13 days (IQR 5–29). ICU readmission occurred in 10.4% of cases. In-hospital mortality was 26.2% (Table 1).

**Table 1.** Characteristics of immediate postoperative patients admitted to the ICU

**Variables**	**Frequency**	**%**
Male sex	126	62.4

Presence of comorbidities	176	87.1
Hypertension	120	59.4
Diabetes mellitus	63	31.2
Cardiopathy	62	30.7
Cancer	39	19.3
Chronic kidney disease	24	11.9
Stroke	19	9.4
Chronic obstructive pulmonary disease	18	8.9
Peripheral vascular disease	16	7.9
Hypothyroidism	15	7.4
HIV/AIDS	4	2.0
Liver cirrhosis	4	2.0

Habits and addictions		
Smoking	81	40.1
Alcohol consumption	46	22.8
Illicit drug use	6	3.0

Surgical priority		
Urgent	106	52.5
Elective	96	47.5

Surgical complexity		
Major	140	69.3
Moderate	47	23.3
Minor	15	7.4

Contamination potential		
Clean	78	38.6
Potentially contaminated	54	26.7
Contaminated	47	23.3
Infected	23	11.4

Type of anesthesia		
General anesthesia	151	74.8
Neuraxial anesthesia	33	16.3
Combined anesthesia	18	8.9

Surgical procedure		
Digestive system, accessory organs, and abdominal wall surgery	52	25.7
Central and peripheral nervous system surgery	41	20.3
Genitourinary system surgery	28	13.9
Circulatory system surgery	23	11.4
Thoracic surgery	21	10.4
Oncologic surgery	15	7.4
Musculoskeletal system surgery	10	5.0
Other surgeries	4	4.0
Minor surgeries and surgeries involving skin, subcutaneous tissue, and mucosa	2	1.0
Endocrine gland surgery	2	1.0
Ocular system surgery	2	1.0
Oral and maxillofacial surgery	2	1.0

ICU: Intensive care unit; HIV: Human immunodeficiency virus; AIDS: Acquired immunodeficiency syndrome.

The main characteristics of the surgical procedures were urgent status, major complexity, and clean classification. The most common type of anesthesia was general anesthesia. The most frequent procedures involved the digestive system, associated organs and abdominal wall, followed by surgeries of the central and peripheral nervous system and the genitourinary system (Table 1).

The preoperative fasting time was documented in 64 patients, ranging from a minimum of 273 to a maximum of 1,500 minutes. Preoperative fasting was longer in non-survivors (a median of 855 minutes, IQR 556–1110) than in survivors (a median of 610 minutes, IQR 541–880; p = 0.0290). Early postoperative feeding was initiated in 34.2% of cases (Table 2). Delays in the timing of postoperative feeding initiation were associated with a higher frequency of complications (p = 0.001).

**Table 2:** Bivariate analysis of risk factors for postoperative complications in patients admitted to the ICU

	**Total**	**Without complications**	**With complications**	**p**
Postoperative feeding				
Up to 6 hours	69 (34.2%)	19 (27.5%)	50 (72.5%)	0.0001*
6 to 12 hours	41 (20.3%)	8 (19.5%)	33 (80.5%)	
12 to 24 hours	47 (23.3%)	2 (4.3%)	45 (95.7%)	
24 to 48 hours	12 (5.9%)	1 (8.3%)	11 (91.7%)	
> 48 hours	16 (7.9%)	0	16 (100.0%)	
Did not occur	17 (8.4%)	0	17 (100.0%)	

Intraoperative fluid resuscitation				
≤ 30 mL/Kg	134 (76.1%)	24 (17.9%)	110 (82.1%)	0.3613
> 30 mL/Kg	42 (23.9%)	5 (11.9%)	37 (88.1%)	

Fluid resuscitation in the IPO				
≤ 30 mL/Kg	59 (92.2%)	6 (10.2%)	53 (89.8%)	0.4574
> 30 mL/Kg	5 (7.8%)	0	5 (100.0%)	

Intraoperative NV prophylaxis				
No	77 (38.1%)	6 (7.8%)	71 (92.2%)	0.0272
Yes	125 (61.9%)	24 (19.2%)	101 (80.8%)	

PO NV prophylaxis				
No	8 (4.0%)	1 (12.5%)	7 (87.5%)	0.8490
Yes	194 (96.0%)	29 (14.9%)	165 (85.1%)	

PO: Postoperative; IPO: Immediate postoperative period; NV: Nausea and vomiting; PO; NV: Postoperative nausea and vomiting; ICU: Intensive care unit. Chi-square test for trend.

**Fig. 2.**
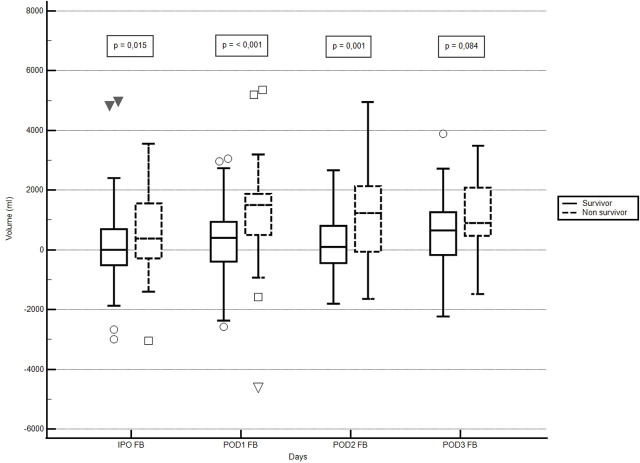
**Comparison of fluid balance in patients during the postoperative period in the ICU.** Legend: ICU – intensive care unit; IPO – immediate postoperative period; POD1 – postoperative day 1; POD2 – postoperative day 2; POD3 – postoperative day 3; FB – fluid balance.

Intraoperative fluid resuscitation was performed in 95.2% of patients. Survivors received a higher volume of crystalloids (median 1,350 mL, IQR 950–2,000) than non-survivors (median 1,000 mL, IQR 500–1,750; p = 0.0265). Fluid resuscitation in the intraoperative and immediate postoperative periods was ≤ 30 mL/kg in most patients, without any significant difference between those with and without complications (Table 2). Fluid balance was higher in non-survivors than in survivors during the immediate postoperative period (IPO), postoperative day 1 (POD1), and postoperative day 2 (POD2) (Figure 2).

Nausea and vomiting prophylaxis were administered to 61.9% of patients intraoperatively and to 96% postoperatively. Patients who received intraoperative prophylaxis experienced fewer complications than those who did not (p = 0.027) (Table 2).

Postoperative complications occurred in 84.7% of patients—79.2% among survivors and 100% among non-survivors. The most frequent complications were cardiovascular (53.4%), infectious (49.5%), and gastrointestinal (48.5%). Although surgical complications were not the most common, they were still present in 37.1% of patients. Other postoperative complications included renal (28.7%), respiratory (18.8%), electrolyte (12.4%), neurological (10.9%), and coagulation-related complications (4.0%).

The most frequent cardiovascular complications were shock (77.8%), arrhythmias (21.3%), and deep vein thrombosis (9.2%). Among the infectious complications, the most common sites were pneumonia (PNM) (36%) and urinary tract infection (UTI) (34%). Septic shock was the initial presentation in 42% of infectious cases.

The most common gastrointestinal complication was prolonged or paralytic ileus, occurring in 43.9% of patients, followed by feeding intolerance (30.6%), postoperative vomiting (17.3%), and the need for parenteral nutrition (PN) (11.2%). The most frequent surgical complications were reoperation (58.7%), need for postoperative blood transfusion (44%), anastomotic leakage or dehiscence (34.7%), and major bleeding (26.7%).

Renal complications were associated with a higher risk of death than any other complication, increasing the risk of death by 14 times. Coagulation-related complications ranked second, followed by cardiovascular, electrolyte, infectious, and respiratory complications (Table 3).

**Table 3.** Bivariate analysis of postoperative complications as a risk factor for death in patients admitted to the ICU in the immediate postoperative period

**Complications**	**OR**	**95% CI**	**p**
Renal (N = 58)	14.09	6.68 – 29.72	< 0.0001
Coagulation system (N = 08)	9.38	1.83 – 48.06	0.0072
Cardiovascular (N = 108)	4.80	2.29 – 10.04	< 0.0001
Electrolyte (25)	4.64	2.16 – 9.97	0.0001
Infectious (N = 100)	4.01	2.01 – 8.03	0.0001
Respiratory (N = 41)	2.16	1.02 – 4.55	0.0424
Neurological (N = 28)	2.13	0.85 – 5.34	0.1033
Surgical (N = 75)	1.59	0.84 – 3.00	0.1541
Gastrointestinal (N = 92)	1.02	0.55 – 1.92	0.9268

OR: Odds ratio; CI: Confidence interval; ICU: Intensive care unit.

In the logistic regression analysis, female sex, urgent surgery, and SAPS 3 score within the first hour of ICU admission were identified as independent risk factors for in hospital mortality (Table 4).

**Table 4.** Logistic regression model for the analysis of risk factors for death in patients admitted to the ICU in the immediate postoperative period

	**Bivariate**			**Multivariate**		
Variable	OR	95% CI	p	OR	95% CI	p
Complications	27.30	1.63 – 454.98	0.0212			
Urgent surgery	8.11	3.57 – 18.42	< 0.001	3.43	1.39 – 8.49	0.0074
SOFA in the IPO	1.22	1.12 – 1.32	< 0.001			
SAPS 3	1.07	1.05 – 1.10	< 0.001	1.07	1.04 – 1.10	< 0.0001
Age	1.01	0.99 – 1.03	0.1039			
Comorbidities	0.77	0.31 – 1.89	0.5744			
Male sex	0.72	0.38 – 1.36	0.3134	0.37	0.16 – 0.85	0.0204

OR: Odds ratio; CI: Confidence interval; SAPS 3: Simplified Acute Physiology Score 3; SOFA: Sequential Organ Failure Assessment; IPO: Immediate postoperative period; ICU: Intensive care unit. Overall model fit: Chi-square = 2.68; Hosmer–Lemeshow test = 0.95; Area under the ROC curve = 0.848 (95% CI: 0.791–0.8955).

## Discussions

The results show that postoperative complications were common among patients admitted to the ICU. Renal and coagulation-related complications showed the strongest association with death. Abbreviation of fasting was associated with a lower incidence of complications. In addition, fluid balance was higher in non-survivors.

The clinical profile of the patients included in this study was similar to that reported in other studies analyzing surgical patients admitted to ICUs [13,14]. Literature data support that postoperative recovery in older adults is slower than in younger patients, which increases the risk of developing complications and death, especially when comorbidities are also present [15].

Early postoperative feeding along with nausea and vomiting prophylaxis showed a positive impact on patient outcomes. A meta-analysis demonstrated that early feeding in gynecologic patients is safe, promotes the return of gastrointestinal peristalsis, reduces the risk of infectious complications and hospital length of stay, and increases patient satisfaction [16]. A European cohort study conducted in 71 centers showed that low tolerance to early feeding was the main independent risk factor for postoperative complications, unplanned reoperations, and longer hospital stays [17].

Although fluid resuscitation was administered restrictively in most patients, it was not associated with a reduction in complications. However, larger volumes of fluid resuscitation were associated with in hospital mortality, as reported by other authors [18]. Perhaps the best way to assess the impact of fluids on patient outcomes is by analyzing fluid balance during the intraoperative and immediate postoperative periods. A study conducted in Brazil with 479 patients undergoing major surgeries found that non-survivors had a higher fluid balance, which was associated with a longer hospital stay and an increased risk of complications [19]. Some of these findings are similar to those of our study, in which non-survivors had a higher fluid balance than survivors. The findings of this study suggest that limiting fluid resuscitation was not sufficient to impact mortality, but that maintaining a controlled fluid balance was associated with better outcomes. A strategy that includes other components of fluid balance may have positive results in the survival of these patients, as it has been shown that resuscitation fluids represent a small component of fluid intake in critically ill patients [20].

Our results indicate that postoperative complications were frequent among patients admitted to the ICU, and most of them were associated with a higher risk of death, especially renal and coagulation-related complications. These findings are consistent with those of other authors, who reported postoperative complications in 57% of patients [21].

Cardiovascular complications were the most frequent, followed by infectious, gastrointestinal, and surgical complications. A recent Latin American study involving lower-risk surgical patients identified infections as the most common complications, which were also associated with increased mortality [22]. Other authors have described cardiovascular complications as the most prevalent postoperative complications [13,14,23,24], including those occurring in the post-anesthesia care unit, where they were directly related to case severity and a higher risk of death [25]. Most of these complications involved ischemic cardiac events, shock requiring vasoactive drugs, and arrhythmias, which may triple the risk of stroke [23].

Although less frequent in this study, renal complications were also associated with increased hospital morbidity and mortality, even in patients who achieved full recovery of renal function [14,23]. Overall, postoperative complications were associated with a reduction in patient survival by 69% and may have a greater impact than preoperative risk assessment or intraoperative factors [26].

The multivariate analysis identified female sex, SAPS 3 score, and urgent surgeries as independent risk factors for in-hospital death. Despite the predominance of males in the study population, male sex was not a significant predictor of death. Gender variable was analyzed within a multivariate model with other variables relevant to death outcome, but the present study’s results regarding women having a higher risk of death contradict existing literature [13,14]. Although the multivariate model resulted in a good, calibrated model with good fit according to ROC curve and Hosmer-Lemeshow test, it is possible that it lacks variables that could detect a result consistent with other authors.

Urgent surgeries were associated with a higher likelihood of death, similar to the findings of the SCORIS study [13]. Patients undergoing this type of procedure often require immediate intervention without sufficient time for proper preparation and clinical optimization before surgery, which increases the risk of mortality. The SAPS 3 prognostic score emerged as an independent risk factor in the multivariate analysis, showing strong discriminatory power for predicting mortality. This score is recognized as a good predictor of mortality in critically ill surgical patients [14].

Regarding in-hospital mortality, there are significant differences when comparing high-income countries to middle- and low-income countries. A cohort study analyzing mortality rates in 28 European countries identified high rates in four countries: Poland, Latvia, Romania, and Ireland (17.9%, 21.5%, 6.8%, and 11.2%, respectively), compared to the United Kingdom (3.6%) [27]. The in-hospital mortality rates reported in Brazilian ICUs were lower than those found in the present study, with rates of 8.9% and 15% reported in the literature [5,11]. Patients undergoing urgent surgeries had even higher rates (17.86%) than those undergoing elective surgeries (5.05%) [28].

Factors that may explain the mortality rates observed in this study include the fact that the sample consisted solely of patients admitted to the ICU in the postoperative period. In addition, the high proportion of urgent surgeries and the patients’ clinical status at admission—many of whom had been transferred from other healthcare facilities with greater clinical complexity—may also have contributed.

Among this study’s limitations is the fact that despite the high number of complications, this variable was included in the multivariate analysis as a single variable, and it was not possible to include each type of complication individually due to the sample size. Moreover, data collection based on medical records may have been incomplete due to missing documentation. As a single-center and retrospective study, it also has inherent limitations, especially limitations in generalizing the results when compared with international data.

Despite the wide range of studies addressing the characteristics of critically ill patients and specific surgical populations, few studies in South America have comprehensively described surgical patients and their complications. This study’s sample allowed for the evaluation of several types of complications and the identification of those associated with a higher risk of death.

## Conclusion

This study found a high incidence of postoperative complications, with renal and coagulation-related ones being the most strongly associated with death. Intra-operative nausea and vomiting prophylaxis and early postoperative feeding were associated with a lower frequency of complications. The identified risk factors for in-hospital death were female sex, SAPS 3 score, and urgent surgeries.
